# Integration of single-cell RNA-sequencing and machine learning identifies GRN and FCER1G as potential peroxisomal targets in influenza pathogenesis

**DOI:** 10.1186/s12879-026-13514-0

**Published:** 2026-05-25

**Authors:** Ning Shan, Shibin Chen, Zhaoyu Liu, Zhe Wen, Junwei Wang, Yao Yu, Xining Liu, Shangwei Ning, Hong Chen

**Affiliations:** 1 Department of Pulmonary and Critical Care Medicine, Second Affiliated Hospital of Harbin Medical University, Harbin, China; 2 College of Bioinformatics Science and Technology, Harbin Medical University, Harbin, China

**Keywords:** Peroxisome, Influenza, Classical monocyte, Single-cell RNA sequencing, Machine learning, *GRN*, *FCER1G*

## Abstract

**Background:**

Influenza, an acute respiratory infection caused by influenza viruses, imposes a significant burden on the healthcare system. Peroxisomes have been shown to be associated with various viral infections, including influenza virus infections, but the specific mechanisms of their involvement in influenza virus infection remain to be explored.

**Methods:**

In this re-analysis of publicly available single-cell and bulk transcriptomic datasets, single-cell scoring algorithms, including AUCell, UCell, singscore, ssgsea, and AddModuleScore, were used to determine the expression of peroxisome-related genes at the single-cell level. Differential gene expression and protein–protein interaction (PPI) analyses identified key peroxisome-related genes. Four machine learning methods, including XGBoost, Boruta, LASSO, and random forest, were integrated to identify optimal characteristic genes.

**Results:**

We observed heterogeneity in the expression of peroxisome-related genes in different cell types during influenza, with non-classical monocytes and classical monocytes having the highest gene expression scores. Compared with those in the control group, classical monocytes had higher scores in the influenza group. Through machine learning algorithms, *GRN* and *FCER1G* were identified as optimal characteristic genes, and their differential expression and diagnostic value for influenza were verified in bulk datasets, with upregulation in classical monocytes.

**Conclusions:**

This study reveals the associations of *GRN* and *FCER1G* with peroxisomes in influenza and the heterogeneity of peroxisome-related genes at the single-cell level during influenza. Additionally, classical monocytes may play a crucial role in the functional realization of peroxisomes in the context of influenza. Our research enhances the understanding of the role of peroxisomes in influenza virus infection and may point to potential therapeutic targets for influenza.

**Supplementary Information:**

The online version contains supplementary material available at 10.1186/s12879-026-13514-0.

## Introduction

Influenza, which is caused by influenza viruses, is an acute respiratory viral infection that gives rise to approximately one billion seasonal cases each year [1]. Among these cases, the number of those falling into the severe category ranges from three to five million, resulting in between 290,000 and 650,000 respiratory fatalities each year [1]. This places a substantial burden on the global health system.

Peroxisomes exhibit remarkable plasticity, enabling them to alter their properties when faced with cellular or environmental stimuli [2]. Their functional contributions encompass a range of metabolic activities in mammalian cells, spanning lipid metabolism and the synthesis and clearance of reactive oxygen species (ROS) and reactive nitrogen species (RNS) [3–5]. Furthermore, peroxisomes have been demonstrated to perform key functions within the innate immune response directed at pathogens, including viruses and bacteria, as well as in the regulation of inflammatory responses [6–8].

Recently, emerging research has underscored the importance of peroxisomes in viral infections. Current research indicates that peroxisomes are involved in regulating signal transduction pathways that prompt host cells to produce interferons (IFNs), interferon-stimulated genes (ISGs), and proinflammatory cytokines after viral infection, thereby inhibiting viral replication and limiting infection [9]. However, studies have indicated that influenza A virus (IAV) may disrupt peroxisomal lipid metabolism by downregulating peroxisomal β-oxidation and promoting the enrichment of ether-linked phosphatidylcholines [10, 11]. This interference subsequently facilitates the efficient assembly of viral particles [11]. There is also an association among influenza virus infection, peroxisomes, and programmed cell death. A study by Jiang et al. revealed that pexophagy induced by several viruses, including the H9N2 avian influenza virus, disrupts the redox homeostasis maintained by peroxisomes, thereby promoting ferroptosis in cells [12]. As a key platform for the cellular antiviral response, peroxisomes can be regulated by viruses in the context of viral infection, which is conducive to the formation and spread of viral particles and thus has dual antiviral and virus-promoting roles [11].

Although recent research has underscored the importance of peroxisomes in influenza virus infection, the identification of their key regulatory genes still poses a challenge. Single-cell RNA sequencing (scRNA-seq) surpasses the limitations of traditional methods in the examination of individual cells and makes it possible to uncover cellular heterogeneity [13]. Machine learning algorithms can be employed to analyze bulk datasets and screen out key regulatory genes. Combining scRNA-seq with machine learning techniques allows for more efficient identification of potential diagnostic biomarkers.

However, within the context of the “dual role”, the target sites and biomarkers of peroxisomes during influenza virus infection remain largely elusive. In this study, scRNA-seq and bulk RNA-seq datasets were integrated to explore peroxisome-associated genes in influenza. We also aimed to identify their primary effector cells. Subsequently, machine learning algorithms were utilized for screening the genes most closely linked to peroxisomes, and these findings were validated via external datasets. This study provides novel insights into the dual role of peroxisomes in influenza and paves the way for future investigations into their precise mechanisms.

## Methods

### Data acquisition

In the present study, the scRNA-seq dataset GSE149689, along with the bulk datasets GSE111368 and GSE101702, were sourced from the Gene Expression Omnibus (GEO) database (https://www.ncbi.nlm.nih.gov/geo/). The GSE149689 dataset encompasses peripheral blood mononuclear cell (PBMC) samples collected from 11 patients with COVID-19, 5 patients with influenza, and 4 healthy control individuals. For subsequent analysis, all influenza patient samples (*n* = 5; GSM4509013, GSM4509014, GSM4509016, GSM4509017, GSM4509018) and healthy control samples (*n* = 4; GSM4509015, GSM4509023, GSM4509024, GSM4509029) from GSE149689 were selected. The remaining samples in this series correspond to COVID-19 patients and were excluded. The GSE111368 dataset consists of microarray data derived from whole blood samples of 109 influenza patients, including some with follow-up data and 130 healthy controls. Among these, 109 baseline samples from influenza patients and 130 healthy control samples were chosen for further analysis. The GSE101702 dataset is a microarray dataset containing peripheral blood samples from 107 influenza patients and 52 healthy controls. All samples within this dataset were selected for subsequent analysis. The peroxisome-related genes were retrieved from the hallmark gene set “HALLMARK_PEROXISOME” in the Molecular Signatures Database (MSigDB) (https://www.gsea-msigdb.org/gsea/msigdb/human/geneset/HALLMARK_PEROXISOME.html), totaling 104 genes, and are described as “genes encoding components of peroxisome”.

### ScRNA-seq dataset analysis

The scRNA-seq dataset GSE149689 was analyzed via the Seurat package (v5.1.0) in R [14]. A threshold was applied whereby genes must be expressed in at least 3 cells to be retained. The cells were subsequently filtered in accordance with the following criteria to guarantee data quality: 200 to 5000 genes were detected in each cell, the total unique molecular identifier (UMI) of each cell was < 30,000, the expression level of mitochondrial genes was < 25%, the expression level of ribosomal genes was < 45%, and the expression level of hemoglobin genes was < 0.1%. For dimensionality reduction, principal component analysis (PCA) was implemented on the genes with relatively high expression levels, and the top 30 principal components were selected for subsequent analyses. Although all samples in GSE149689 were processed within the same study, each sample—whether from an influenza patient or a healthy control—was generated as an independent biological replicate. Technical sources of variation across samples (e.g., differences in cell viability, library preparation efficiency, and sequencing depth) can introduce systematic biases known as batch effects. To integrate cells across multiple samples while minimizing this technical noise, we applied the RunHarmony function from the harmony package (v1.2.3). Harmony projects cells into a shared low-dimensional embedding where cells group by cell type rather than by sample of origin, thereby enabling robust comparisons between influenza and healthy control groups. The FindClusters function was subsequently utilized for clustering, and the resolution was set at 1.0. The RunUMAP function was applied to draw the uniform manifold approximation and projection (UMAP) plot. Cell type annotation was performed by referencing the marker genes which were derived from the original literature, CellMarker 2.0, and PanglaoDB [15–17].Key markers included *CD79A* and *MS4A1* for B Cells, *CD8A* and *CD8B* for CD8 + T Cells, *IL7R* and *CCR7* for CD4 + T Cells, *GNLY* and *KLRD1* for NK Cells, *CD14* and *CD93* for classical monocytes, *FCGR3A* and *CDKN1C* for nonclassical monocytes, *HBB* for erythrocytes, *PPBP* and *PF4* for platelets.

### Scoring of peroxisome-related genes

We employed 5 algorithms to compute the scores of peroxisome-related genes in each cell, namely, “AUCell”, “UCell”, “singscore”, “ssgsea”, and “AddModuleScore“ [14, 18–21]. AUCell utilizes the area under the curve (AUC) to measure the activity of the gene set in each cell. UCell computes the gene set activity in each cell via the Mann‒Whitney U method. Singscore analyzes the expression of the gene set through a method based on the ranking of gene expression quantities. Ssgsea calculates the cumulative enrichment score of genes within the gene set by sorting and normalizing the gene expression values of each individual cell. AddModuleScore obtains a score by calculating the average expression value of genes in the gene set in each cell. For each cell, the five algorithm scores were first min–max normalized and then averaged to obtain a composite peroxisome-related gene score. For each annotated cell type, the composite peroxisome-related gene scores were compared between the influenza and healthy control groups. Depending on the normality of score distribution, either an unpaired t-test or a Wilcoxon rank-sum test was employed.

### Differential gene expression and correlation analysis

Cells were then dichotomized into high- and low-score groups based on the median of these composite scores across all cells. Then, DEGs associated with peroxisomal components were identified via the FindMarkers function, with |logFC| > 1 and an adjusted p value < 0.05 used as the significance criteria when contrasting high- and low-expressing groups. In addition, a correlation analysis was performed to identify the genes closely related to the components of the peroxisomes, and the top 100 genes ranked by the correlation coefficient were selected. Consequently, the intersection of the upregulated DEGs and the top 100 genes from the correlation ranking was taken for subsequent analysis.

### Protein‒protein interaction network

To select genes with more intimate interactions, we delineated a protein‒protein interaction (PPI) network. The intersected genes obtained were entered into the STRING database (https://cn.string-db.org/), and disconnected nodes within the network were excluded. The PPI network was subsequently constructed via Cytoscape software (v3.10.3), and nodes with a degree of ≥ 10 were chosen for subsequent analysis.

### Enrichment analysis

To determine the functions of the genes corresponding to these proteins with degrees ≥ 10 and their potential mechanisms involved in the onset of influenza and the regulation of peroxisome components, we performed Gene Ontology (GO) enrichment analysis by employing the R package clusterProfiler (v4.8.3) [22].

### Identification of optimal characteristic genes via machine learning

Four machine learning algorithms, eXtreme Gradient Boosting (XGBoost) (https://CRAN.R-project.org/package=xgboost), Boruta, least absolute shrinkage and selection operator (LASSO), and random forest (RF) (https://cran.r-project.org/web/packages/randomForest/), were utilized to screen the optimal characteristic genes associated with peroxisomes [23, 24]. XGBoost enhances its robustness by iteratively training decision trees, where each iteration corrects the errors of the previous iteration. In our study, feature importance was evaluated using permutation-based importance with 10 permutations. Genes were ranked by descending average importance across all permutations, and an elbow-point criterion was applied to the ranked importance scores to retain the most influential features. The Boruta algorithm, which is grounded in the random forest framework, automatically identifies features that make significant contributions to the prediction target. This is accomplished by evaluating the importance of genuine features against a set of randomly created shadow features, which enhances the model’s performance and interpretability. In our study, the Boruta algorithm was configured with 500 trees. Only features with importance statistically significantly higher than the maximum shadow feature importance across multiple iterations were classified as Confirmed and retained. LASSO improves the accuracy and interpretability of the prediction model by integrating regularization terms into the loss function. In our study, the LASSO algorithm with 10-fold cross-validation was employed to guarantee the model’s robustness and prevent overfitting. The optimal regularization parameter lambda.1se was selected, and genes with non-zero coefficients at this penalty level were retained. The RF algorithm evaluates feature importance by constructing multiple decision trees and aggregating their prediction results through classification or averaging algorithms. In our research, the RF algorithm was configured with 200 trees. Feature importance was ranked by Mean Decrease Gini, and an elbow-point criterion was applied to the ranked importance scores to retain the top contributors. All machine learning algorithms were implemented on samples from baseline influenza patients and healthy controls in GSE111368. The results were subsequently summarized via an UpSet plot, and the intersection of the four algorithms was identified as the optimal characteristic genes for the role of peroxisomes in influenza.

### The expression and predictive significance of optimal characteristic genes in influenza blood samples

Depending on the normality of the data distribution, either the t test or the Wilcoxon rank-sum test was employed to assess the expression of the optimal characteristic genes in the GSE111368 and GSE101702 datasets. Moreover, in these two datasets, receiver operating characteristic (ROC) curves of the optimal characteristic genes were constructed, and the area under the curve (AUC) was calculated. These served as internal and external validations for the results of the machine-learning analysis, respectively.

### Verification of optimal characteristic genes at the single-cell level

Validation of optimal characteristic gene expression in single-cell data was performed by analyzing the scRNA-seq dataset GSE149689. This analysis aimed to evaluate the enrichment of these genes at the single-cell level, with the ultimate goal of verifying the specific cell types that play a role in the contribution of peroxisome components to the occurrence of influenza.

To further evaluate the clinical relevance of *GRN* and *FCER1G*, we stratified influenza patients in both datasets according to disease severity. In the training dataset GSE111368, patients were classified based on the requirement for respiratory support (including mask/nasal cannula oxygen supplementation and mechanical ventilation): 62 patients did not require respiratory support and 47 required respiratory support. In the validation dataset GSE101702, influenza patients were grouped into severe and non-severe cases according to the clinical metadata provided by the original study. For each severity stratification, gene expression levels were compared between groups, and ROC curves were generated to assess discriminatory performance. Confusion matrices were constructed using the optimal cutoff determined by the Youden index to calculate sensitivity and specificity.

### Pseudo-time analysis

The cells were classified into two groups according to the expression levels of the optimal characteristic genes. The monocle package (v2.28.0) was used for pseudo - time analysis [25]. The Monocle2 algorithm was applied to reconstruct the developmental paths of specific cellular components. The subset used for pseudo-time analysis comprised all classical monocytes from influenza patients and healthy controls in the GSE149689 dataset. From this subset, scaled UMI counts are extracted to deduce the developmental trajectories of cells. We leveraged pseudo-time analysis to compare the alterations in the expression levels of the optimal characteristic genes in the corresponding cell types of influenza patients and healthy control samples as the pseudo-time progressed.

### Cell communication

To probe intercellular communication, we interrogated signaling networks with the CellChat package (v1.6.1) using the standard CellChatDB for ligand-repair assignment [26]. By identifying the ligands or receptors that are overexpressed in specific cell types, we deduced the interactions both within cell types and between different cell types.

### Annotation scheme and cross-validation

Our primary cell type annotation followed the scheme employed by Choi et al., which is also listed on the GSE149689 GEO page [15]. This scheme focuses on major immune cell lineages and does not separately annotate dendritic cells (DCs) or intermediate monocytes. In our clustering results, no distinct cluster corresponding to CD14⁺ CD16⁺ intermediate monocytes was resolved, consistent with their low abundance in peripheral blood. To ensure that our findings are robust to the choice of annotation strategy, we performed a parallel analysis using Lee et al.’s annotation scheme that includes DCs as a distinct population [27]. A small cluster of *FCER1A*-expressing dendritic cells (264 cells, 0.98% of total) was identified and re-assigned accordingly. Subsequently, we applied the same single-cell scoring and statistical comparison procedures as described in the preceding section “Scoring of peroxisome-related genes”.

## Results

### ScRNA-seq analysis identifies cell populations in peripheral blood samples from influenza patients and healthy controls

**Fig. 1:**
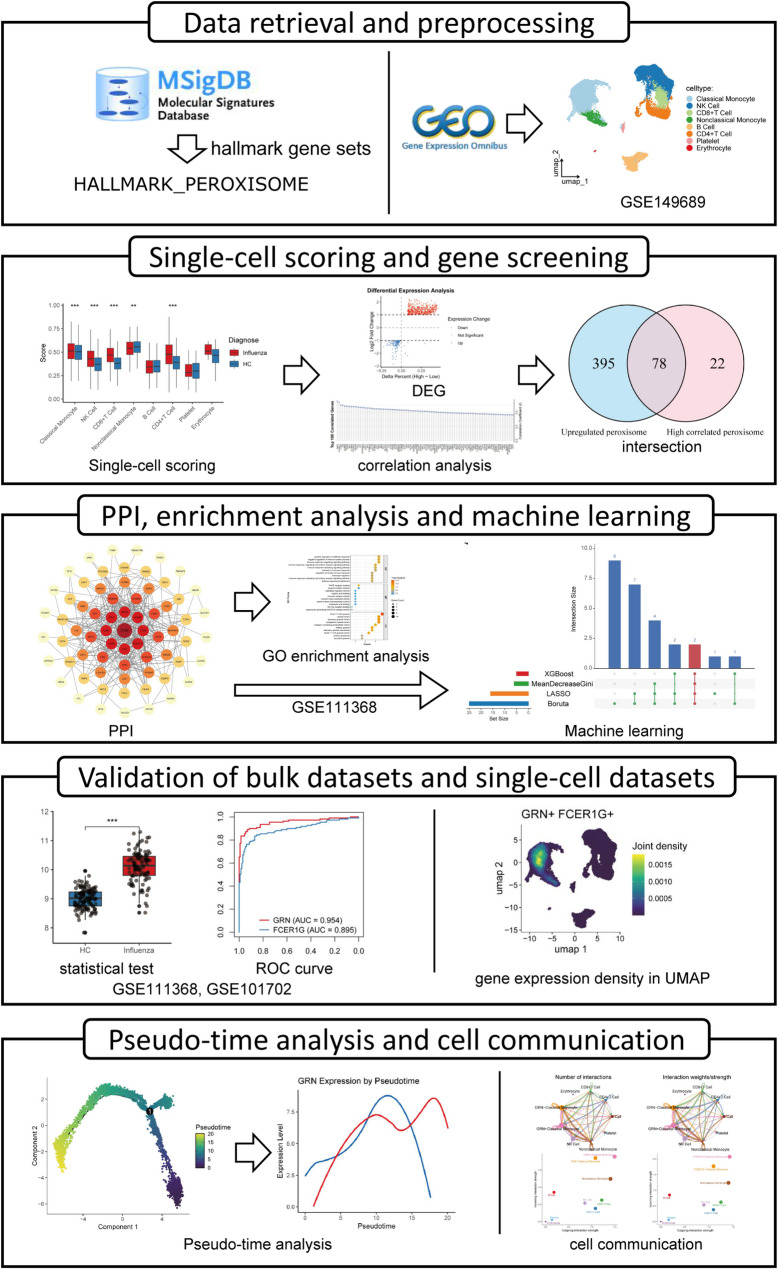
Flowchart of the research design

Figure 1 depicts the flowchart of this study.

**Fig. 2:**
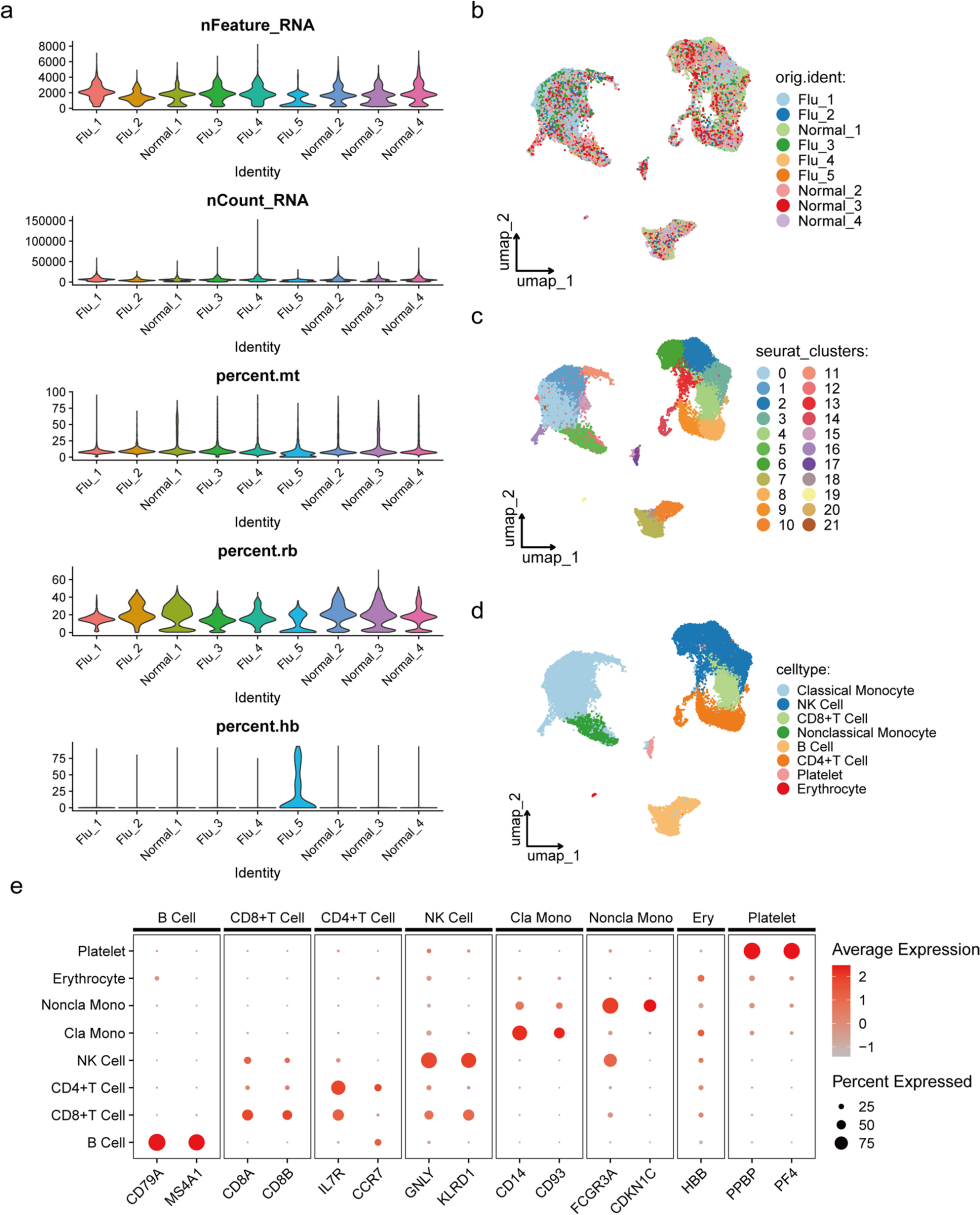
Annotation of cell subpopulations. (**a**) Quality control of single-cell data. (**b**) UMAP display, where batch effects between samples have been removed. (**c**) UMAP plot showing that all cells are classified into 22 clusters. (**d**-**e**) Based on marker genes, the data were manually annotated into 8 different cell types

Initially, data quality control was carried out. After the influenza and healthy control samples from the GSE149689 dataset were selected, quality control was implemented in five aspects, as per the information in Fig. 2a. These aspects included the number of genes detected per cell, UMI counts, and the expression levels of mitochondrial genes, ribosomal genes, and hemoglobin genes. In total, 27,064 high-quality cells were retained. The 9 samples were subsequently analyzed. Batch effects were eliminated, and a UMAP plot was generated (Fig. 2b). The cells were evenly distributed among the samples, indicating the successful correction of batch effects. Following the Seurat pipeline analysis, all the cells were classified into 22 clusters (Fig. 2c). Figure 2d shows the cell types annotated on the basis of marker genes, such as classical monocytes, natural killer (NK) cells, and CD8 + T cells. The expression patterns of the marker genes in each cell type are presented in a dot plot (Fig. 2e).

### The scRNA-seq scoring algorithm identifies classical monocytes along with potentially significantly associated genes

**Fig. 3:**
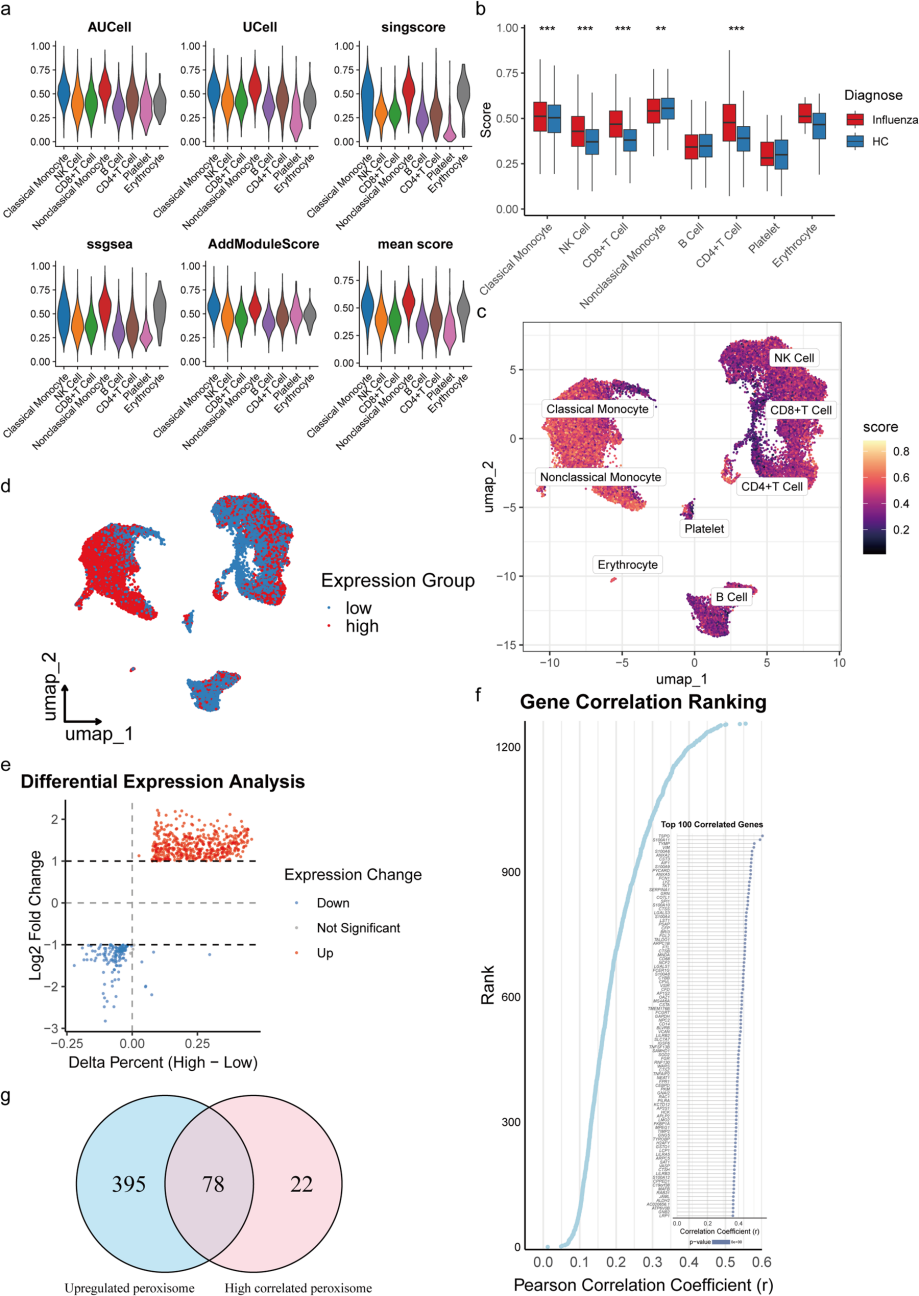
Heterogeneity analysis of peroxisome-related genes expression in influenza. (**a**) Violin plots showing the expression scores of peroxisome-related genes in various cell types calculated via the AUCell, UCell, singscore, ssGSEA, and AddModuleScore algorithms. (**b**) Box plots showing the score differences between the influenza and HC groups in each cell type. *p < 0.05, **p < 0.01, and ***p < 0.001. (**c**) UMAP analysis results indicating that peroxisome-related genes are highly expressed in classical monocytes. (**d**) UMAP of all cells were divided into high-score and low-score groups on the basis of the median peroxisome-related gene score. (**e**) The percentage difference (delta represents the percentage of cells) and log-fold change in the expression of the DEGs between the high-expression and low-expression groups were obtained via the FindMarkers function. (**f**) Correlation analysis was used to select genes significantly correlated with the average score of peroxisome-related genes. (**g**) Venn diagram of the results of the DEGs and correlation analysis

To explore the activity of peroxisomes at the single-cell level in the context of influenza, we employed a suite of algorithms, including AUCell, UCell, singscore, ssgsea, and AddModuleScore, to quantify the expression scores of genes associated with peroxisome components for each individual cell. The results indicated that there was heterogeneity among the scores of different types of cells, nonclassical monocytes exhibited the highest composite peroxisome scores, followed by classical monocytes (Fig. 3a). Comparison between influenza patients and healthy controls revealed that classical monocytes, NK cells, CD8⁺ T cells, and CD4⁺ T cells all showed significantly higher scores in the influenza group, whereas non-classical monocytes showed significantly lower scores (Fig. 3b). Among these significantly altered populations, classical monocytes were unique in satisfying both criteria of a high baseline score and significant upregulation in influenza, and were therefore selected as the primary effector cell population for in-depth downstream analyses. Further analysis via UMAP confirmed the increased activity of peroxisomes in classical monocytes (Fig. 3c). For deeper analysis, we dichotomized all cells into high-score and low-score subsets with the median of the average expression values as the threshold. This stratification revealed that the high-score group was predominantly composed of classical monocytes (Fig. 3d). DEG analysis between the high-score and low-score groups revealed a total of 473 upregulated genes and 145 downregulated genes (Fig. 3e). Additionally, correlation analysis revealed the 100 genes most strongly associated with the peroxisome-related gene scores (Fig. 3f). Notably, 78 genes were found to be present in both the set of upregulated differentially expressed genes and the correlation analysis (Fig. 3g), highlighting their potential significance in the context of peroxisome activity in influenza.

### Cross-validation confirms that the core findings are independent of the annotation strategy

To confirm that our findings were not dependent on the choice of annotation strategy, we repeated the peroxisome scoring analysis using a more granular annotation that includes DCs as a distinct population (Fig. S1a–b). Under this scheme, DCs exhibited the highest composite peroxisome-related gene scores among all immune cell types, followed by non-classical monocytes and classical monocytes (Fig. S1c). Comparison between influenza patients and healthy controls revealed that classical monocytes, NK cells, CD8⁺ T cells, and CD4⁺ T cells had significantly higher scores in the influenza group, whereas non-classical monocytes showed significantly lower scores (Fig. S1d). DCs scores did not differ significantly between groups. Crucially, classical monocytes remained the cell population with significantly elevated peroxisome-related gene activity in influenza patients, consistent with the findings obtained under our primary annotation scheme. These results confirm that our core conclusion is robust to the choice of annotation strategy.

### Integrated screening methods reveal *GRN* and *FCER1G* as optimal feature genes

**Fig. 4:**
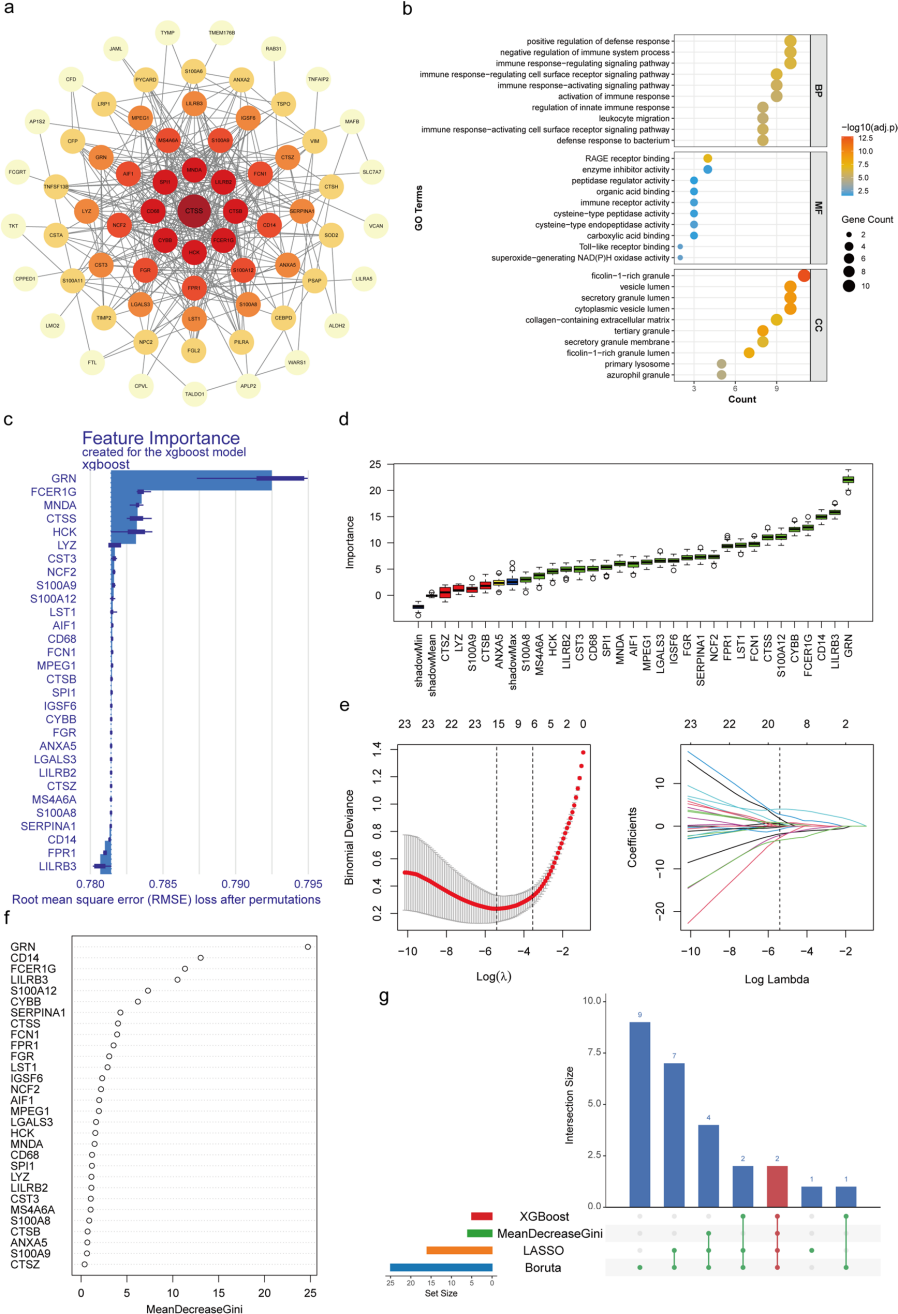
PPI network, GO enrichment, and machine learning screening of feature genes. (**a**) PPI involving 69 proteins. (**b**) GO enrichment analysis of genes corresponding to proteins with a degree ≥ 10. Identification of optimal feature genes via machine learning methods. (**c**-**e**) XGBoost (**c**), Boruta (**d**), LASSO (**e**), and RF (**f**) were used to determine the best optimal feature genes. (**g**) The UpSet plot shows the intersection of the results from the four machine learning methods

After inputting the protein names of the 78 genes mentioned above into the STRING database, excluding the disconnected nodes in the network and importing the results into Cytoscape software, we obtained a PPI containing 69 proteins, which indicated strong interactions among these proteins (Fig. 4a). After selecting proteins with a degree ≥ 10, we obtained 30 proteins with stronger interactions and conducted GO analysis via their corresponding gene names. The 10 genes with the highest gene ratios in each of the 3 biological process (BP), molecular function (MF), and cellular component (CC) categories and with p.adjust < 0.05 were plotted in a dot plot (Fig. 4b, Additional file 1). The results of the GO analysis suggested that these 30 genes were related to multiple biological processes, especially immune responses, which indicated a potential mechanism of peroxisome involvement in the pathogenesis of influenza.

Four machine learning algorithms, namely, XGBoost (Fig. 4c), Boruta (Fig. 4d), LASSO (Fig. 4e), and RF (Fig. 4f), were utilized to screen for candidate feature genes that exhibited the highest correlation with influenza in the training dataset GSE111368. By taking the intersection of the feature genes selected by these 4 algorithms, two optimal characteristic genes were determined: *GRN* and *FCER1G* (Fig. 4g).

**Fig. 5:**
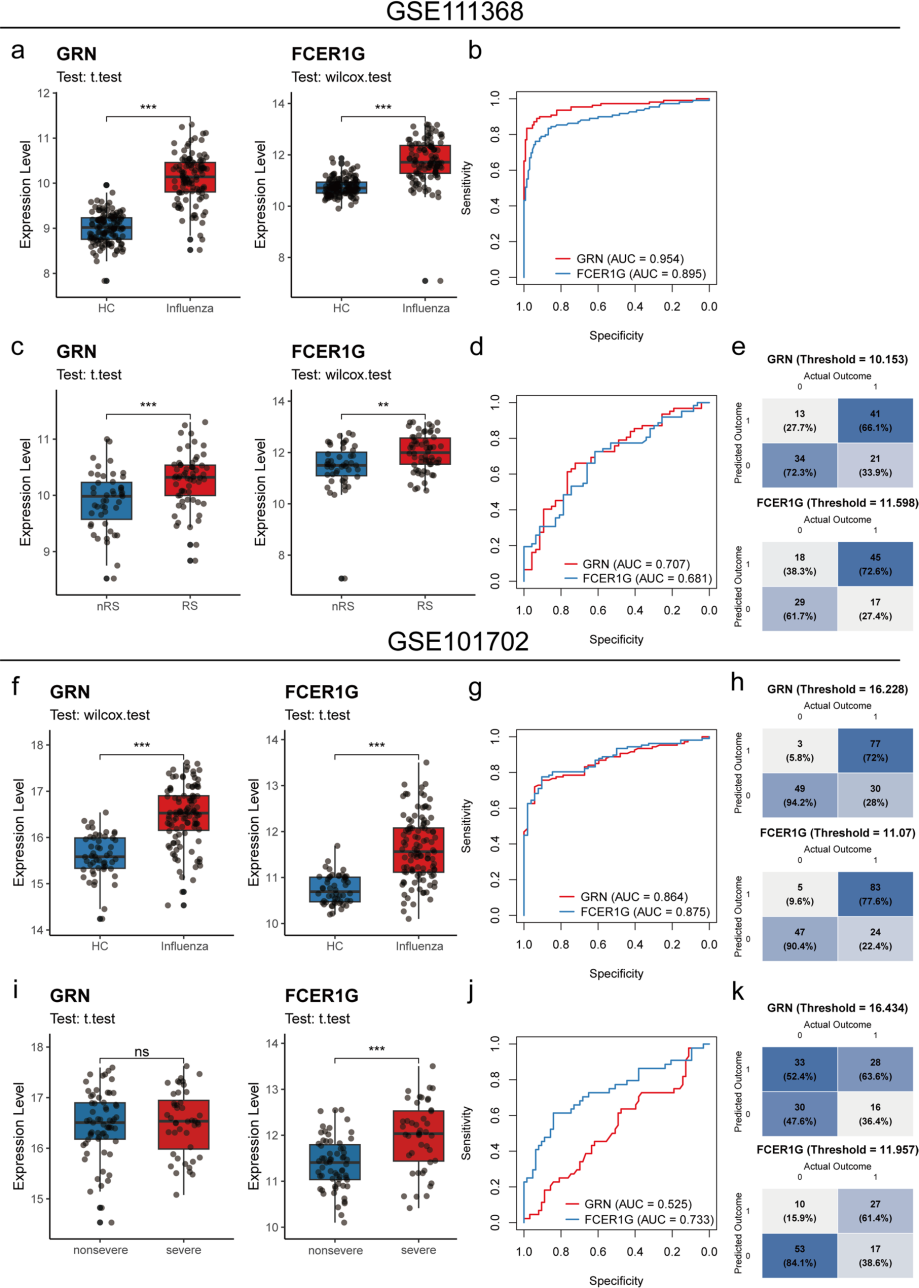
Validation results of optimal feature genes at the bulk level. (**a**) The expression levels of *GRN* and *FCER1G* in the influenza group in the training set were significantly greater than those in the HC group. (**b**) ROC curves evaluating the diagnostic value of *GRN* and *FCER1G* for distinguishing influenza patients from HC in the training set. (**c**) Expression levels of *GRN* and *FCER1G* in the training set stratified by requirement for respiratory support. (**d**) ROC curves for discriminating patients requiring respiratory support in the training set. (**e**) Confusion matrices for respiratory support discrimination in the training set. (**f**) Expression levels of *GRN* and *FCER1G* in the validation set (GSE101702) comparing the influenza group versus HC. (**g**) ROC curves evaluating the diagnostic value of *GRN* and *FCER1G* for influenza in the validation set. (**h**) Confusion matrices for influenza diagnosis in the validation set. (**i**) Expression levels of *GRN* and *FCER1G* in the validation set stratified by disease severity. (**j**) ROC curves for discriminating severe influenza in the validation set. (**k**) Confusion matrices for severe influenza discrimination in the validation set. *p < 0.05, **p < 0.01, and ***p < 0.001. nRS: no need for respiratory support; RS: respiratory support

To assess the diagnostic accuracy of these findings, we first examined the expression levels of *GRN* and *FCER1G* in the training dataset GSE111368 and the validation dataset GSE101702. Both genes were significantly upregulated in the peripheral blood of influenza patients compared with healthy controls (Fig. 5a, f). ROC curve analysis yielded high AUC values in both cohorts: in the training set, the AUCs were 0.954 for *GRN* and 0.895 for *FCER1G* (Fig. 5b); in the validation set, the AUCs were 0.864 for *GRN* and 0.875 for *FCER1G* (Fig. 5g). Furthermore, confusion matrices constructed using the Youden index-derived optimal cutoff showed that in the validation set, *GRN* achieved a sensitivity of 72.0% and a specificity of 94.2% for diagnosing influenza, while *FCER1G* achieved a sensitivity of 77.6% and a specificity of 90.4% (Fig. 5h).

To further evaluate the clinical relevance of *GRN* and *FCER1G*, we stratified influenza patients according to disease severity. In the training dataset GSE111368, patients requiring respiratory support exhibited significantly higher expression of both *GRN* and *FCER1G* than those not requiring respiratory support (Fig. 5c). The AUCs for discriminating patients requiring respiratory support were 0.707 for *GRN* and 0.681 for *FCER1G* (Fig. 5d), with sensitivities of 66.1% and 72.6%, and specificities of 72.3% and 61.7%, respectively (Fig. 5e). In the validation dataset GSE101702, *FCER1G* expression was significantly higher in severe influenza patients than in non-severe cases, whereas *GRN* did not differ significantly between groups (Fig. 5i). The AUCs for discriminating severe influenza were 0.525 for *GRN* and 0.733 for *FCER1G* (Fig. 5j). The sensitivity and specificity for identifying severe cases were 63.6% and 47.6% for GRN, and 61.4% and 84.1% for FCER1G (Fig. 5k). These results indicate that while both peroxisome-related genes have robust diagnostic value for distinguishing influenza patients from healthy controls, their ability to stratify disease severity is moderate, with *FCER1G* showing a more consistent association with severe illness than *GRN*.

### Single-cell level validation confirmed the expression of *GRN* and *FCER1G* in classical monocytes

**Fig. 6:**
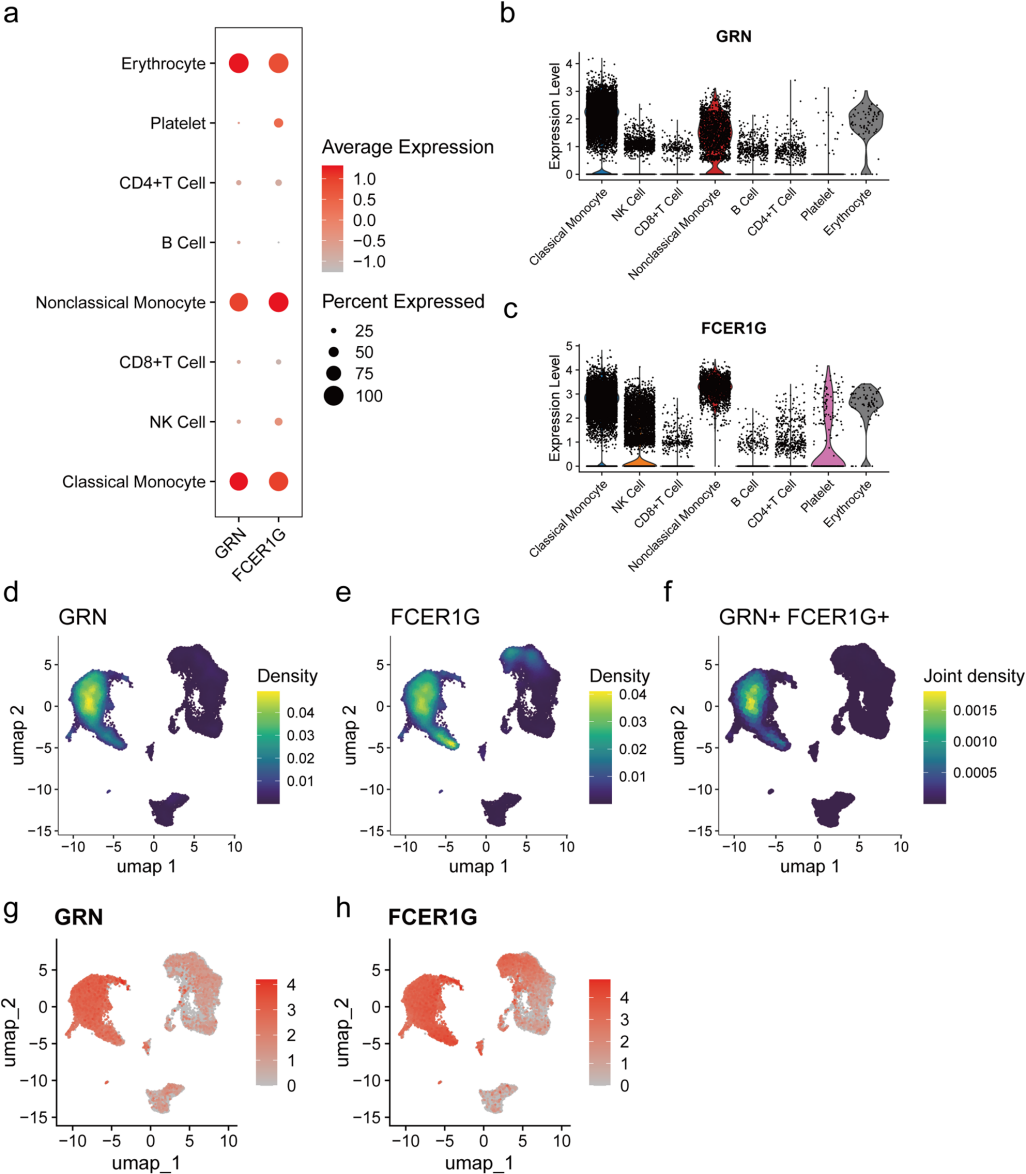
Validation results of optimal feature genes at the single-cell level. (**a**) Dot plot showing the expression of *GRN* and *FCER1G* in different cell types. (**b**) Violin plot showing the expression of the *GRN* in different cell types. (**c**) Violin plot showing the expression of *FCER1G* in different cell types. (**d**) The gene expression density of the *GRN* is presented in UMAP. (**e**) The gene expression density of *FCER1G* is presented in UMAP. (**f**) The combined gene expression density of the *GRN* and *FCER1G* is presented in UMAP. (**g**-**h**) Gene expression levels of GRN (**g**) and FCER1G (**h**) presented in UMAP

We identified the optimal feature genes of the peroxisome components in the scRNA-seq data. In immune cells, *GRN* and *FCER1G* were expressed mainly in classical and nonclassical monocytes (Fig. 6a-e, g, h). Additionally, compared with nonclassical monocytes, classical monocytes were the cell population in which *GRN* and *FCER1G* were coexpressed (Fig. 6f).

### Pseudo - time analysis and cell–cell communication analysis revealed the potential of *GRN* and *FCER1G* in classical monocytes in the pathogenesis of influenza

**Fig. 7:**
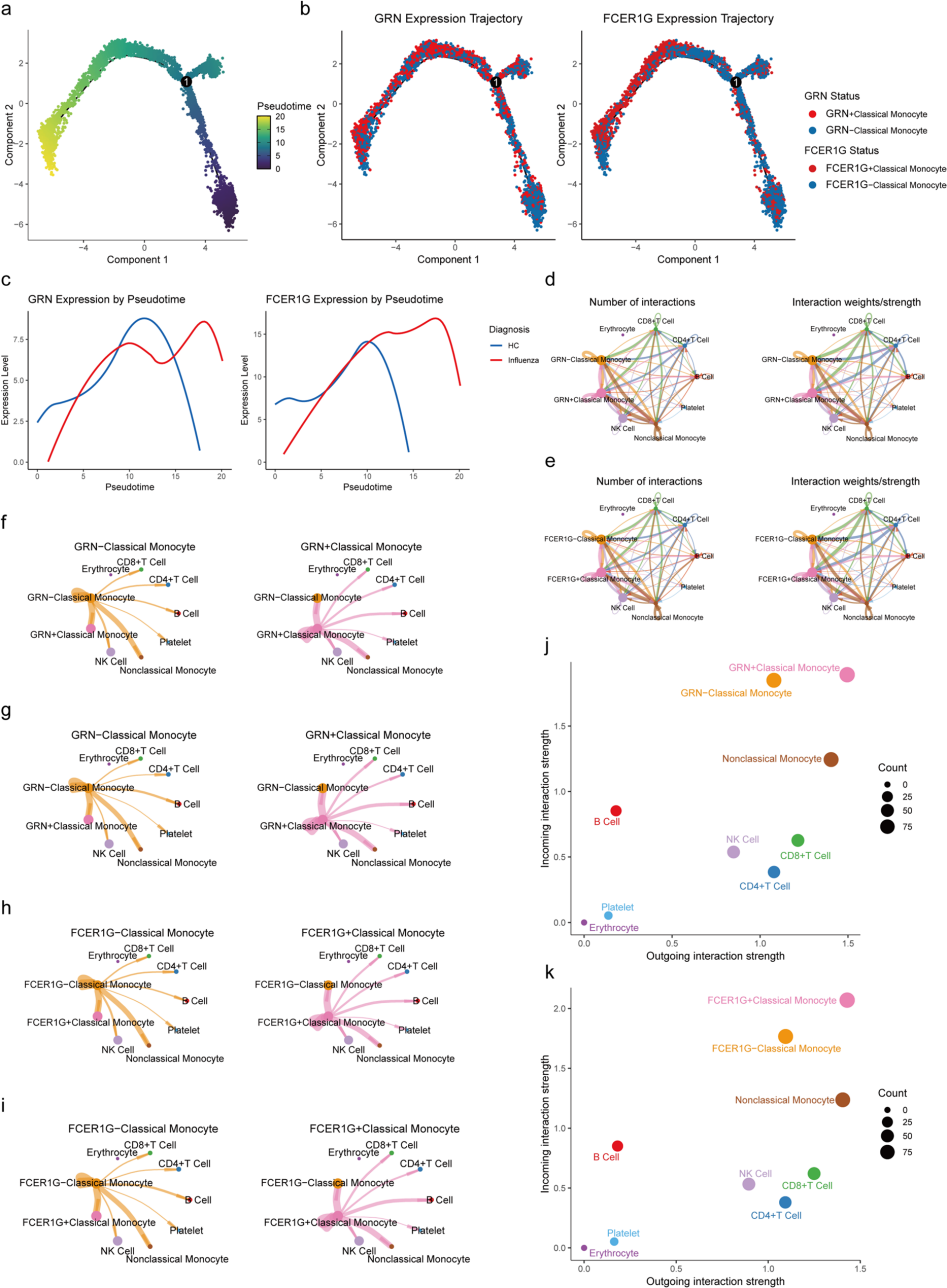
Cell trajectory analysis and cell communication. (a-b) Differentiation process of classical monocytes (**a**), *GRN*+/-, and *FCER1G*+/- (**b**) classical monocytes during the pseudo-temporal process. (**c**) Dynamic changes in the expression levels of *GRN* and *FCER1G* in classical monocytes during the pseudo-temporal process. (**d**-**e**) Circular plots showing the number and strength of interactions between *GRN* + and *GRN*- classical monocytes (**d**), *FCER1G* + and *FCER1G*- classical monocytes (**e**) and other cell types. Different colors in the circular plots represent different cell types, and the width of the edge is proportional to the interaction number or weight between the shown cells. (**f**-**g**) The number (**f**) and strength (**g**) of interactions between *GRN*+/- classical monocytes and other cell types. (**h**-**i**) The number (**h**) and strength (**i**) of interactions between *FCER1G*+/- classical monocytes and other cell types. (**j**-**k**) Relationships between the differential outgoing interactions and incoming interaction strengths between *GRN* + and *GRN*- classical monocytes (**j**) and between *FCER1G* + and *FCER1G*- classical monocytes (**k**)

To explore the transcriptional heterogeneity of classical monocytes pseudo-time, we performed an analysis using Monocle2 (Fig. 7a). During the quasitemporal process, the proportions of *GRN*+ classical monocytes and *FCER1G*+ classical monocytes gradually increased as the pseudo-temporal sequence progressed (Fig. 7b). The pseudo - time analysis also revealed the relative expression patterns of *GRN* and *FCER1G* between the influenza and HC groups. Overall, the expression of both genes gradually increased in the influenza samples as the pseudo - time sequence progressed and was greater than that in the HC samples (Fig. 7c). Cell communication analysis revealed the number and strength of interactions between *GRN*+, *GRN*-, *FCER1G* + and *FCER1G*- classical monocytes and other cells (Fig. 7d-i). Compared with *GRN*- and *FCER1G*- classical monocytes, *GRN* + and *FCER1G*+ classical monocytes had greater incoming and outgoing interaction strengths (Fig. 7j, k).

## Discussion

Influenza constitutes a substantial threat to the life and health of the global population. Although new guidelines and medications are constantly emerging, the high incidence and mortality rates during susceptible seasons still represent challenges that we must confront [1, 28].

Research on the mechanism through which influenza viruses infect host cells and induce pathological alterations has remained unceasing in recent years. There has been a notable increase in recent studies addressing the function of peroxisomes in viral infections, including influenza [9, 29–31]. Peroxisomes were initially identified in the renal tubular epithelial cells of mice by J. Rhodin in 1954 and were designated as “microbody“ [32]. In 1966, De Duve and colleagues successfully isolated peroxisomes and elucidated their biochemical properties; within this organelle, several enzymes responsible for generating hydrogen peroxide and degrading it were identified; consequently, De Duve and colleagues named it the “peroxisome“ [32]. Peroxisomes are ubiquitously present in diverse cell types of eukaryotes; they carry out various functions (such as fatty acid α- and β-oxidation, ether phospholipid biosynthesis, and glyoxylate detoxification) and safeguard cells by oxidizing substrates via the oxidases present within them and hydrolyzing the by-product hydrogen peroxide generated during this process via catalase [9].

Upon infection of host cells by viruses, viral RNA is released into the cytoplasm and recognized by retinoic acid-inducible gene I (RIG-1)-like receptors, such as RIG-I, or by melanoma differentiation-associated protein 5 (MDA-5) [9]. Peroxisomes serve as the key signaling platform for cellular antiviral immunity, on which the mitochondrial antiviral signaling protein (MAVS) recognizes viral RNA through its interaction with RIG-I and MDA-5 [33]. Following this recognition, MAVS is activated and recruited to peroxisomes to initiate a signaling cascade that leads to the activation of the transcription factors interferon regulatory factor (IRF)1 and IRF3, ultimately resulting in the production of type I and III interferons (IFNs) and IFN-stimulated genes (ISGs) to mediate the antiviral response [6, 9, 34].

Some studies have suggested that IAV infection can induce the downregulation of peroxisomal fatty acid β-oxidation and the enrichment of peroxisome-derived ether-linked phosphatidylcholines relative to ester-linked phosphatidylcholines and that the impairment of β-oxidation can increase susceptibility to influenza A virus [10, 35]. In addition, peroxisomal biogenesis factor 19 (PEX19) can interact with IAV to inhibit IAV proliferation; however, if the peroxisome pool is reduced, it promotes the replication of IAV [31].

It should be noted that the present analysis examines peripheral blood mononuclear cells, which reflect the systemic host immune response to respiratory influenza infection, rather than virus-infected cells at the site of infection in the respiratory tract. The transcriptomic signatures observed in circulating immune cells may therefore represent downstream effects of inflammatory mediators and systemic immune activation, rather than direct virus–host interactions within infected cells.

Our research utilized a combination of scRNA-seq and machine learning algorithms to analyze the complex role of peroxisomes during influenza virus infection at the cellular level. We identified *GRN* and *FCER1G* as optimal characteristic genes closely related to peroxisomes in influenza and found that these genes are located mainly in classical monocytes. These findings highlight the regulatory role of these genes and classical monocytes in peroxisome function during influenza virus infection and the human antiviral immune response. This advanced approach enabled us to reveal the role and function of peroxisomes in influenza virus infection at the cellular and molecular levels. To ensure the robustness of the analysis, we validated the expression of these genes and their diagnostic value for influenza in both internal and external datasets. The identification of these genes provides new insights into the role of peroxisomes in influenza virus infection and can serve as potential therapeutic targets.

The granulin precursor (*GRN*), the protein progranulin (PGRN) encoded by this gene, has a molecular structure characterized by containing 7.5 highly conserved 12-cysteine granulin/epithelin motif repeat sequences [36]. Under the hydrolytic action of proteases, PGRN can release multiple independent active GRN protein fragments [37, 38]. The roles of the GRN and PGRN in various biological processes have been widely studied, and their main functions include host defence, inflammatory response, lysosomal storage disease, frontotemporal dementia, etc [39–42]. GRN has a proinflammatory effect, whereas PGRN has anti-inflammatory activity [43–45]. During influenza virus infection, the activity of neutrophil elastase increases, which can promote the conversion of PGRN to GRN, thereby aggravating the inflammatory response [45]. Interestingly, Luo et al. reported that PGRN is significantly upregulated in the plasma of pediatric patients with H1N1 influenza, and animal experiments indicate that PGRN aggravates pulmonary immunopathological damage during influenza virus infection [46]. This seems to contrast with the anti-inflammatory activity of PGRN. We speculate that this might be due to the increased PGRN leading to a higher level of the transformed GRN. Intriguingly, PGRN, a protein with anti-inflammatory activity, can be converted into a proinflammatory GRN. This relationship is in line with the dual functions of peroxisomes, which are both antiviral and proviral. We hypothesize that, in the context of influenza virus infection, the manifestation of this “dual function” of peroxisomes may be intricately associated with the dynamic regulation of PGRN-to-GRN conversion.

*FCER1G* (Fc epsilon receptor Ig) encodes the Fc receptor γ-chain (FcRγ). This protein forms a dimer on the cell membrane and serves as an essential component of multiple immunoglobulin receptors, including high-affinity Fc epsilon receptor I (FcεRI), the IgG receptors Fc gamma receptor I (FcγRI) and Fc gamma receptor IIIA (FcγRIIIA), and the IgA receptor Fc alpha receptor I (FcαRI) [47]. Among these, FcεRI is the central receptor mediating the effector functions of IgE and is involved in allergic responses [47]. FcRγ contains an immunoreceptor tyrosine-based activation motif (ITAM) in its intracellular domain and functions as a signaling adaptor within the FcεRI complex [47]. As the most abundant immunoglobulin in the circulation, IgG interacts via its Fc region with FcγRI and FcγRIIIA. This engagement triggers antibody-dependent cellular cytotoxicity (ADCC) and antibody-dependent cellular phagocytosis (ADCP), thereby coordinating the recognition and clearance of pathogens [48]. In monocytes, FcγRI primarily activates ADCP and contributes to mediating ADCC in both monocytes and macrophages [49, 50]. Notably, FcγRIIIA (CD16/FCGR3A) is a marker for non-classical monocytes and is expressed at low levels on classical monocytes. Studies in mouse models further underscore the critical role of FcRγ in antiviral immunity: its ablation leads to a loss of both ADCC and ADCP functions, indicating its indispensable role in antibody-mediated immune clearance [51]. In our GO analysis, genes containing *FCER1G* were enriched in BP components such as immune response-regulating signaling pathway, immune response-activating signaling pathway, immune response-regulating cell surface receptor signaling pathway, activation of immune response, and immune response-activating cell surface receptor signaling pathway. These findings indicate that *FCER1G* is widely involved in various immune pathways and that its specific molecular-level interaction mechanisms with influenza virus and peroxisomes are worthy of further study.

Interestingly, Li et al. reported that the expression of *FCER1g* was significantly upregulated in *PGRN* gene knockout mice after influenza virus infection, suggesting that *PGRN* can regulate the expression of *FCER1g* [52]. In our pseudo-temporal analysis, between 10 and 15 on the x-axis, there was a decrease in *GRN* expression and an increase in *FCER1G* expression in the influenza samples. Within this temporal range, we observed the same trend as that reported by Li et al. However, in other temporal ranges, the expression trends of *GRN* and *FCER1G* were the same, which is contrary to the findings of Li et al. We speculate that the possible reasons for this discrepancy could be species differences, selection bias, or the fact that the study did not cover the entire post-infection period.

Another important finding is that peroxisome-related gene scores are heterogeneous among different cell types, with the highest scores in non-classical monocytes and classical monocytes. Among them, the influenza score of classical monocytes was greater than that of healthy monocytes, suggesting the role of classical monocytes in influenza virus infection and postinfection immunity. As a subset of monocytes, classical monocytes are characterized by CD14 + CD16 (FCGR3A) expression, which is opposite to that of non-classical monocytes (CD14- CD16+); this can be reflected in our cell annotation. In the cynomolgus macaque model, classical monocytes in PBMCs bind to A549 cells expressing HA in an FcγR-dependent manner, highlighting the role of classical monocytes and the expression of the *FCER1G* gene in the protection of primate anti-influenza immunity [53]. The heterogeneity of peroxisome-related gene expression in different cells provides new insights into the role of peroxisomes in influenza and highlights the correlation between classical monocytes and peroxisomes, which can serve as potential therapeutic targets.

To address the clinical relevance of *GRN* and *FCER1G*, we further examined their association with influenza severity using the available clinical indicators in each dataset. In the training cohort (GSE111368), patients were stratified by the requirement for respiratory support; both *GRN* and *FCER1G* expression levels were significantly higher in patients requiring respiratory support. In the validation cohort (GSE101702), patients were stratified into severe and non-severe groups according to the clinical metadata; the association with severe disease was significant for *FCER1G* but not for *GRN*. The moderate AUC values for severity stratification (0.681–0.733 for *FCER1G*; 0.525–0.707 for *GRN*) indicate that while these genes may reflect aspects of disease severity, their primary clinical utility lies in diagnostic discrimination rather than prognostic stratification. Future studies with larger cohorts and more granular clinical metadata are warranted to fully evaluate their prognostic value.

Our research also has several limitations. First, the data of this study were derived from public datasets, which may lead to bias in patient selection. To minimize these biases, we employed multiple scoring systems and machine learning algorithms. Second, this is a purely computational study based on re-analysis of publicly available transcriptomic data. The associations identified between *GRN*/*FCER1G*, peroxisome-related gene sets, and influenza are statistical correlations, and the conclusions drawn from these analyses should be interpreted within the context of computational prediction. Third, as this study relies entirely on computational analyses, the identified associations await experimental characterization. Further investigation through in vitro and in vivo experiments would help clarify whether *GRN* and *FCER1G* directly participate in peroxisomal regulation during influenza infection. Fourth, our study did not cover the entire cycle after influenza virus infection. Future research conducted throughout the entire post-infection period would be valuable to reveal the dynamic changes in the antiviral and proviral functions of peroxisomes. Future research should focus on clarifying the specific molecular pathways by which *GRN* and *FCER1G* regulate peroxisome and classical monocyte functions in the context of influenza.

## Conclusion

Our research revealed that the *GRN* and *FCER1G* are associated with peroxisomes in the context of influenza, providing new insights into the role of peroxisomes during influenza virus infection. Furthermore, their significant upregulation in patients requiring respiratory support or with severe disease suggests a potential role in reflecting disease severity, particularly for *FCER1G*, which showed a consistent association across independent cohorts. Our study also highlighted the role of classical monocytes in peroxisome function in the context of influenza. These genes not only enhance our understanding of the role of peroxisomes in influenza virus infection and may point to different therapeutic targets and future research directions.

## Supplementary Information

Below is the link to the electronic supplementary material.

Supplementary Material 1

Supplementary Material 2

## Data Availability

The datasets generated and/or analyzed during the current study are available in the GEO repository, https://www.ncbi.nlm.nih.gov/geo/query/acc.cgi?acc=GSE149689, https://www.ncbi.nlm.nih.gov/geo/query/acc.cgi?acc=GSE111368, https://www.ncbi.nlm.nih.gov/geo/query/acc.cgi?acc=GSE101702, and MSigDB, https://www.gsea-msigdb.org/gsea/msigdb/human/geneset/HALLMARK_PEROXISOME.html.

## Abbreviations

ADCC: Antibody-dependent cellular cytotoxicity
ADCP: Antibody-dependent cellular phagocytosis
AUC: Area under the curve
BP: Biological process
CC: Cellular component
DEG: Differentially expressed gene
FCER1G: Fc epsilon receptor Ig
FcRγ: Fc receptor γ-chain
FcαRI: Fc alpha receptor I
FcγRI: Fc gamma receptor I
FcγRIIIA: Fc gamma receptor IIIA
FcεRI: Fc epsilon receptor I
GEO: Gene Expression Omnibus
GO: Gene Ontology
GRN: Granulin precursor
IAV: Influenza A virus
IFN: Interferon
IRF: Interferon regulatory factors
ISG: Interferon-stimulated gene
ITAM: Immunoreceptor tyrosine-based activation motif
LASSO: Least Absolute Shrinkage and Selection Operator
MAVS: Mitochondrial antiviral signaling protein
MDA-5: Melanoma differentiation - associated protein 5
MF: Molecular function
MSigDB: Molecular Signatures Database
PCA: Principal component analysis
PEX19: Peroxisomal biogenesis factor 19
PGRN: Progranulin
PPI: Protein‒Protein Interaction
RF: Random Forest
RIG-1: Retinoic acid - inducible gene I
RNS: Reactive nitrogen species
ROC: Receiver operating characteristic
ROS: Reactive oxygen species
scRNA-seq: Single-cell RNA sequencing
UMAP: Uniform manifold approximation and projection
UMI: Unique Molecular Identifier
XGBoost: eXtreme Gradient Boosting
