# Supplementary material for: Integration of single-cell RNA-sequencing and machine learning identifies GRN and FCER1G as potential peroxisomal targets in influenza pathogenesis
Source: BMC Infect Dis. 2026 May 25;26:1376. doi: 10.1186/s12879-026-13514-0 (PMC13386947; doi:10.1186/s12879-026-13514-0)
Supplement: Supplementary file 2 — Supplementary Material 2 [file 12879_2026_13514_MOESM2_ESM.docx]

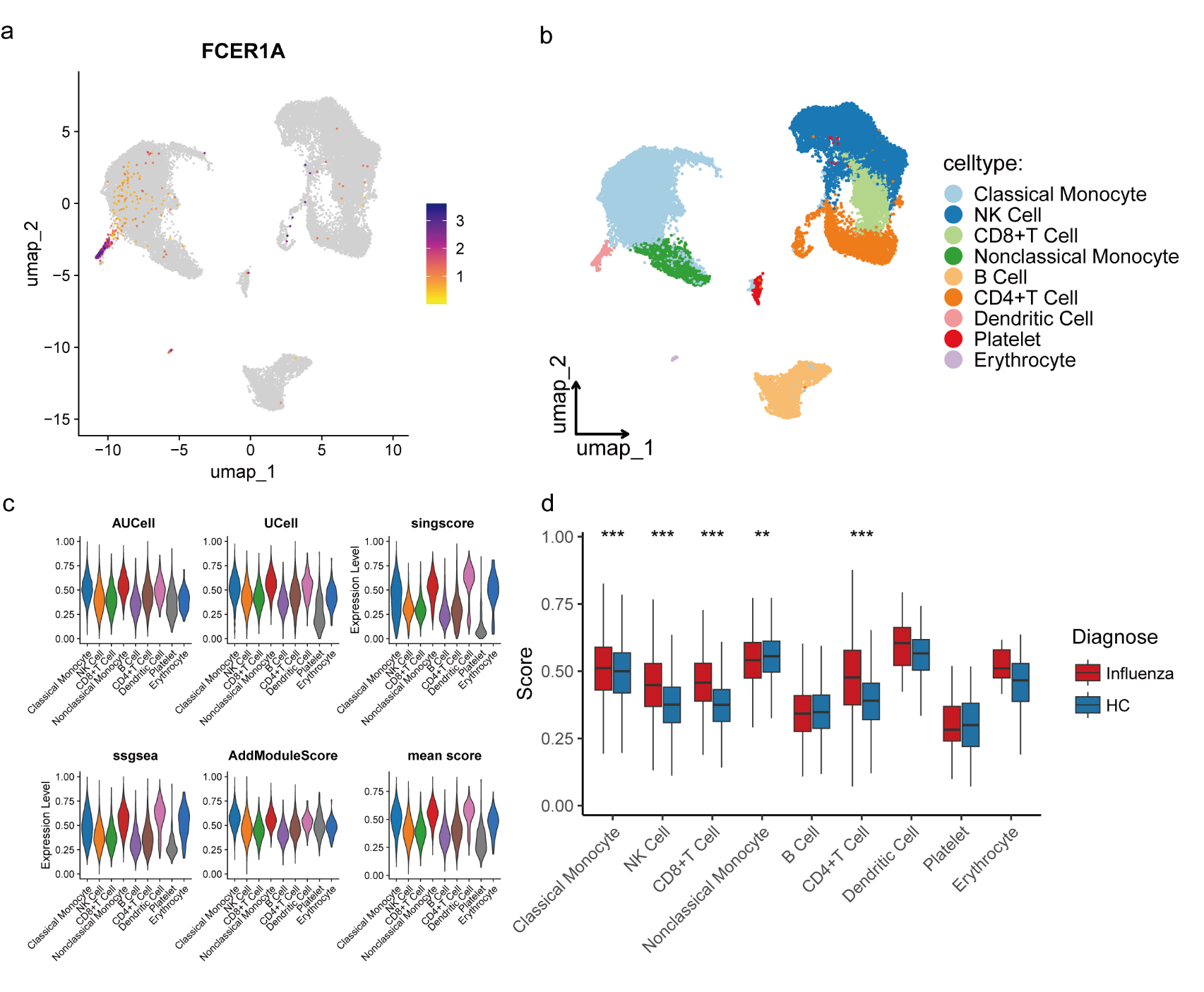


**Figure S1. Cross-validation of peroxisome-related gene scores under the Lee et al. annotation scheme.**
(a) Feature plot showing *FCER1A* expression, identifying the dendritic cell cluster (Seurat cluster 16). (b) UMAP projection with DCs annotated as a distinct cell type. (c) Violin plots of peroxisome-related gene scores (five algorithms and composite score) across all cell types including DCs. (d) Box plots comparing composite peroxisome scores between influenza patients and healthy controls for each cell type. *p < 0.05, **p < 0.01, and ***p < 0.001.
